# *TNFAIP3* Gene Polymorphisms in a Chinese Han Population with Vogt–Koyanagi–Harada Syndrome

**DOI:** 10.1371/journal.pone.0059515

**Published:** 2013-03-21

**Authors:** Hong Li, Qing Liu, Shengping Hou, Liping Du, Qingyun Zhou, Yan Zhou, Aize Kijlstra, Peizeng Yang

**Affiliations:** 1 First Affiliated Hospital of Chongqing Medical University, Chongqing, P.R. China; 2 Eye Research Institute Maastricht, Department of Ophthalmology, University Hospital Maastricht, Maastricht, The Netherlands; The Scripps Research Institute, United States of America

## Abstract

**Background:**

This study was performed to evaluate the potential association of TNFAIP3 polymorphisms with Vogt–Koyanagi–Harada (VKH) disease in a Chinese Han population.

**Methodology/Principal Findings:**

Five single-nucleotide polymorphisms (SNPs), rs10499194, rs610604, rs7753873, rs5029928 and rs9494885 of TNFAIP3 were genotyped in 834 VKH disease patients and 1415 healthy controls using a PCR-restriction fragment length polymorphism assay. An increased frequency of the C allele and CT genotype for rs9494885 were found in VKH patients in the Guangzhou and Chongqing cohorts (pc = 0.015, OR = 1.6, pc = 0.036, OR = 1.7; pc = 2.36×10−4, OR = 1.5, pc = 0.012, OR = 1.5, respectively). Meanwhile, a decreased frequency of the TT genotype for rs9494885 was observed in VKH patients in the Guangzhou and Chongqing cohorts (pc = 0.026, OR = 0.6, pc = 0.0074, OR = 0.7, respectively). The combined analysis showed that a significantly increased prevalence of the rs9494885 TC genotype and C allele were found in VKH disease patients compared with controls (pc = 2.26×10−5, OR = 1.7; pc = 1.09× 10−5, OR = 1.6, respectively). The frequency of the TT genotype of rs9494885 was markedly lower in VKH disease patients as compared with that in controls (pc = 1.12×10−5, OR = 0.6**;** p_c_ = 1.09×10^−5^, OR = 0.6, respectively). No association was found between rs10499194, rs610604, rs7753873 and rs5029928 polymorphisms and VKH disease. To our knowledge this is the first report describing the association of a TNFAIP3 gene polymorphism with VKH disease in a Chinese Han population.

**Conclusions/Significance:**

The results suggest that the rs9494885 TC genotype and C allele may be predisposing factors to VKH disease, whereas the rs9494885 TT genotype and T allele may provide protection against this disease.

## Introduction

VKH disease is one of the most common uveitis entities in China [1]. It is characterized by a granulomatous panuveitis frequently in association with extraocular findings such as pleocytosis in the cerebrospinal fluid (CSF), dysacusis, alopecia, poliosis, and vitiligo [2], [3], [4]. Previous studies have suggested that VKH disease is possibly mediated by a T-cell-mediated autoimmune response directed against melanocytes [5]. A genetic predisposition as a key element in the susceptibility to this disease comes from the observation of several familial cases [6], [7], [8] and an increased prevalence in pigmented racial groups [2]. Furthermore, a strong association between VKH disease and the HLA system (HLA-DR4 and HLA-DRw53) has been reported [9], [10], [11], [12]. Recent studies have shown that polymorphisms of several immune related genes such as cytotoxic T lymphocyte-associated antigen-4 (CTLA-4) [13],interleukin (IL)–17 [14], signal transducers and activators of transcription 4 (STAT4) [15] and osteopontin (OPN) [16] are associated with susceptibility to VKH disease. The exact role of these genetic factors in the pathogenesis of this disease is not well understood and more studies are needed using large sample size in different ethnic populations.

TNFAIP3 encodes a cytoplasmic zinc finger protein, known as the A20 protein. This protein is required for the negative regulation of the NF-KB signaling pathway mediated by innate immune receptors such as TNF receptors and Toll-like receptors, thereby preventing overstimulation of the innate immune response [17], [18]. Several studies have suggested a role for TNFAIP3 polymorphisms in the susceptibility to complex autoimmune disorders, including rheumatoid arthritis (RA) [19], [20], psoriasis [21], systemic lupus erythematosus (SLE) [22], [23], Sjögren’s syndrome (SS) [24], Crohn’s disease [24], and ulcerative colitis [25]. These findings suggest that TNFAIP3 may be a common risk gene for a number of immune-related disorders. In this study, we tested whether TNFAIP3 polymorphisms were associated with the susceptibility to VKH disease in a Chinese Han population. The results showed that rs9494885 was strongly associated with VKH disease.

## Materials and Methods

### Study Population

Eight hundred and thirty four VKH disease patients (male vs female = 464 vs 370, age = 31.6±7.2) and 1415 controls (male vs female = 803 vs 612, age = 32.7±7.3), all belonging to the Chinese Han population, were included in this study. There were no differences in age, sex and ethnicity between patients and controls (p>0.05). The blood samples were obtained from the First Affiliated Hospital, Chongqing Medical University (Chongqing, China) and the Uveitis Study Center of the Sun Yat-sen University (Guangzhou, China). The diagnosis of VKH disease followed the revised criteria for this disease [26]. The clinical characteristics of the VKH disease patients were assessed at the time of diagnosis and are summarized in table 1.

**Table 1:** Clinical features of the investigated VKH disease patients.

Clinical features	Total (n = 834)	%
Age at onset (years±SD)	31.6±7.2	
Male	464	55.6
Female	370	44.4
Headache	445	53.4
Neck stiffness	350	42.0
Dysacusis	198	23.7
Tinnitus	345	41.4
Alopecia	309	37.1
Poliosis	257	30.8
Vitiligo	153	18.3
Scalp hypersensitivity	146	17.5

### Ethics Statement

All the patients and controls participated in this study gave written informed consent. This study was approved by the Clinical Research Ethics Committee of the First Affiliated Hospital of Chongqing Medical University (Permit Number: 2009-201008). All procedures followed the tenets of the Declaration of Helsinki.

### SNP Selection and Genotyping

We studied 5 SNPs including rs10499194, rs610604, rs7753873, rs5029928 and rs9494885 in the TNFAIP3 region on 6q23 which were demonstrated earlier by other groups to be associated with certain immune-related diseases [19], [20]. Genomic DNA was isolated from blood leukocytes using the commercial Qiagen DNA Blood Mini kit (Qiagen, Valencia, CA). The extracted DNA was stored at −20°C until use. Amplification of target DNA was performed by PCR. The primers used in this study are presented in table 2. SNPs were genotyped by restriction fragment length polymorphism analysis. The amplification was performed using initial denaturation at 95°C for 5 minute, 95°C for 30 seconds, 58–62°C for 30 seconds, 72°C for 30 seconds, and 72°C for 5minutes followed by 37 cycles. The PCR products were incubated with restriction enzymes for at least 4 hours. Digestion products were visualized on a 4.0% agarose gel and stained with Goodview (SBS Genetech, Beijing, China). Direct sequencing was performed by Invitrogen Biotechnology Company (Guangzhou, Guangdong province, China) using randomly selected subjects (20% of all samples) to validate the method used in this study.

**Table 2:** Primers and restriction enzymes used for RFLP analysis of the TNFAIP3 gene.

rs number	Primers	Tm(°C)	Restriction enzyme
rs10499194	5′CCACCTTGAATTTCTTAGCTCTG 3′	62	MseI/TRUII
	5′GCGCCACTGCACTCCAAA 3′		
rs610604	5′TCCCCTGCTCGCTGTTTT 3′	60	SacI
	5′GCGCCTTTGAGTGTGTCTGC 3′		
rs7753873	5′ ATGCCTCATTTATTCACTCAAC 3′	60	TSP509I
	5′CCAAAGGGATGCTCTGTC 3′		
rs5029928	5′GGGAGAAGAGTTTGAGTAAC 3′	60	XapI (ApoI)
	5′GCAGCTAAGGCAATGGAG 3′		
rs9494885	5′TACCAGCCACATAGCAAGCA 3′	58	hinfI
	5′CAGGGCATATGTGGGAGAAA 3′		

### Statistical Analysis

Distribution of genotypes and alleles between patients and normal controls was analyzed using SPSS version 17.0 (SPSS, Inc., Chicago, IL). The Chi square test was used to compare allele and genotype distributions. When the low cell counts for genotypes less than 10, the Fisher’s exact test was applied. Bonferroni correction was applied for multiple testing.

## Results

The result showed that five SNPS rs10499194, rs610604, rs7753873 and rs5029928 rs9494885 of TNFAIP3 genetic variants were in Hardy–Weinberg equilibrium in the control group and in the patient group. The distribution and frequencies of genotypes and alleles of the five tested TNFAIP3 polymorphisms are shown in table 3. There were remarkable differences between VKH disease patients and controls concerning rs9494885. An increased frequency of the C allele and CT genotype were found in VKH patients in the Guangzhou and Chongqing cohorts (pc = 0.015, OR = 1.6, pc = 0.036, OR = 1.7; pc = 2.36×10−4, OR = 1.5, pc = 0.012, OR = 1.5, respectively). Meanwhile, a decreased frequency of the TT genotype was observed in VKH patients in the Guangzhou and Chongqing cohorts (pc = 0.026, OR = 0.6, pc = 0.0074, OR = 0.7, respectively). The combined analysis showed that a significantly increased prevalence of the rs9494885 TC genotype and C allele were found in VKH disease patients compared with controls (pc = 2.26×10−5, OR = 1.7; pc = 1.09× 10−5, OR = 1.6, respectively). The frequency of the TT genotype significantly lower in VKH disease patients (pc = 1.12×10−5, OR = 0.6). However, there were no differences in the frequencies of genotypes and alleles of rs610604, rs7753873, rs10499194 and rs5029928 between patients with VKH disease and controls in the Chinese Han population after Bonferroni correction.

**Table 3:** Frequencies of alleles and genotypes of TNFAIP3 polymorphisms in VKH disease patients and controls.

SNP	Genotype allele	Guangzhou Cohort				Chongqing Cohort				Combined Cohorts				
		VKH(%) (N = 272)	Controls (%) (N = 335)	p_c_^b^ Value	OR^a^(95% CI)	VKH(%) (N = 562)	Controls (%) (N = 1080)	p_c_ Value	OR (95% CI)	VKH(%) (N = 834)	Controls (%) (N = 1415)	p_c_ Value		OR (95% CI)
rs10499194	T	13(2.4)	26(3.9)	NS	0.6(0.3–1.2)	41(3.6)	85(3.9)	NS	1.08(0.7–1.6)	54(3.2)	111(3.9)	NS	0.83(0.6–1.0)	
	CC	259(95.2)	309(92.2)	NS	1.7(0.8–3.3)	521(92.7)	995(92.1)	NS	1.09(0.7–1.6)	780(93.5)	1304(92.2)	NS	1.230(0.9–1.7)	
	TC	13(4.8)	26(7.8)	NS	0.6(0.3–1.2)	41(7.3)	85(7.9)	NS	0.9(0.6–1.4)	54(6.5)	111(7.8)	NS	0.813(0.6–1.1)	
	TT	0	0	–	–	0	0	–	–	0	0	–	–	
rs610604	C	50(9.2)	46(6.9)	NS	0.6(0.3–1.2)	102(9.1)	162(7.5)	NS	1.2(0.9–1.6)	152(9.1)	208(7.3)	NS	1.3(1.0–1.6)	
	AA	224(82.4)	289(86.3)	NS	0.7(0.5–1.2)	464(82.6)	921(85.3)	NS	0.8(0.6–1.1)	688(82.5)	1210(85.5)	NS	0.8(0.6–1.0)	
	AC	46(16.9)	46(13.7)	NS	1.3(0.8–2.0)	94(16.7)	156(14.4)	NS	1.2(0.9–1.6)	140(16.8)	202(14.3)	NS	1.2(0.9–1.6)	
	CC	2(0. 7)	0	NS	–	4(0.7)	3(0.3)	NS	2.6(0.6–11.6)	6(0. 7)	3(0. 2)	NS	3.4(0.9–13.7)	
rs5029928	T	25(4.6)	46(6.9)	NS	0.7(0.4–1.1)	64(5.7)	149(6.9)	NS	0.8(0.6–1.1)	89(5.3)	195(6.9)	NS	0.8(0.6–1.0)	
	CC	247(90.8)	290(86.6)	NS	1.5(0.9–2.6)	498(88.6)	932(86.3)	NS	1.2(0.9–1.7)	745(89.3)	1222(86.4)	NS	1.3(1.0–1.7)	
	CT	25(9.2)	44(13.1)	NS	0.7(0.4–1.1)	64(11.4)	147(13.6)	NS	0.8(0.6–1.1)	89(10.7)	191(13.5)	NS	0.8(0.6–1.0)	
	TT	0	1(0. 3)	–	–	0	1(0.1)	–	–	0	2(0.1)	–	–	
rs7753873	C	36(6.6)	46(6.9)	NS	1.0(0.6–1.5)	81(7.2)	143(6.6)	NS	1.1(0.8–1.5)	117(7.0)	189(6.7)	NS	1.1(0.8–1.3)	
	AA	236(86.8)	292(87.2)	NS	1.0(0.6–1.6)	482(85.8)	940(87.0)	NS	0.9(0.7–1.2)	718(86.1)	1232(87.1)	NS	0.9(0.7–1.2)	
	AC	36(13.2)	40(11.9)	NS	1.1(0.7–1.8)	79(14.1)	137(12.7)	NS	1.1(0.8–1.5)	115(13.8)	177(12.5)	NS	1.1(0.9–1.4)	
	CC	0	3(0. 9)	NS	–	1(0.2)	3(0.3)	NS	0.6(0.07–6.1)	1(0. 1)	6(0. 4)	NS	0.3(0.03–2.3)	
rs9494885	C	94(17.3)	76(11.3)	0.015	1.6(1.2–2.3)	152(13.5)	208(9.6)	3.52×10^−3^	1.5(1.2–1.8)	246(14.7)	284(10.0)	1.09×10^−5^	1.6(1.3–1.9)	
	CC	2(0. 7)	1(0. 3)	NS	2.5(0.2–27.4)	7(1.2)	9(0.8)	NS	1.5(0.6–4.0)	9(1.1)	10(0.7)	NS	1.5(0.6–3.8)	
	CT	90(33.1)	74(22.1)	0.036	1.7(1.2–2.5)	138(24.6)	190(17.6)	0.012	1.5(1.2–2.0)	228(27.3)	264(18.7)	2.26×10^−5^	1.6(1.3–2.0)	
	TT	180(66.2)	260(77.6)	0.026	0.6(0.4–0.8)	417(74.2)	881(81.6)	0.0074	0.7(0.5–0.8)	597(71.6)	1141(80.6)	1.12×10^−5^	0.6(0.5–0.7)	

OR^a^: odds ratio;

p_c_^b^: Bonferroni corrected p value.

We further analyzed the relationship between these SNPs and various extraocular clinical findings including headache, neck stiffness, tinnitus, alopecia, poliosis, dysacusis, scalp hypersensitivity, and vitiligo as shown in Table 1. The result showed no relationship of the tested five SNPs with these clinical parameters.

## Discussion

In this study we analyzed the association of five SNPs of the TNFAIP3 gene with susceptibility to VKH disease in a Chinese Han population. The results showed that rs9494885 was strongly associated with VKH disease and that the TT genotype and T allele could provide protection against VKH disease, whereas the TC genotype and C allele were the risk factors for this disease.

VKH disease is an immune-mediated disease and both innate and adaptive immune responses are involved in its pathogenesis [27]. As TNF signaling links the innate and adaptive immune systems, it could help to explain the involvement of both systems in autoimmune diseases. Nowadays the multiple potential roles of TNFAIP3 in regulating autoimmune diseases are not fully understood, and are likely to be cell and context dependent [28], [29], . TNFAIP3,which is required for termination of the nuclear factor-κB (NF-κB) signal that is mediated by innate immune receptors, has been reported to be associated with SLE and rheumatoid arthritis [31]. Several genetic studies have suggested a role for TNFAIP3 in the susceptibility to complex autoimmune disorders. These results prompted us to examine whether the polymorphisms of the TNFAIP3 gene could contribute to the development of VKH disease in a Chinese Han population. In this study, we focused on the association of five SNPs in TNFAIP3 with VKH disease mainly because they were found to be associated with certain autoimmune or rheumatic disease, including psoriasis [21], [32], SLE, RA [20], [23], [33], juvenile idiopathic arthritis [34] and psoriatic arthritis [35]. Although other variants in this gene have also been shown to be associated with autoimmune diseases [19], [36], they were not included in this study due to the fact that these polymorphisms are not present in the Chinese Han population as assessed by an analysis of the HapMap database.

Because the study on the association of gene polymorphisms with disease can be affected by several factors, in this study we made the following efforts to ensure the results. First, all control subjects were matched ethnically and geographically with the patients. Second, we strictly selected the VKH disease patients according to the revised criteria [26]. Third, 20% of the samples were randomly chosen and analyzed by direct sequencing to validate the method employed in this study.

**Figure 1:**
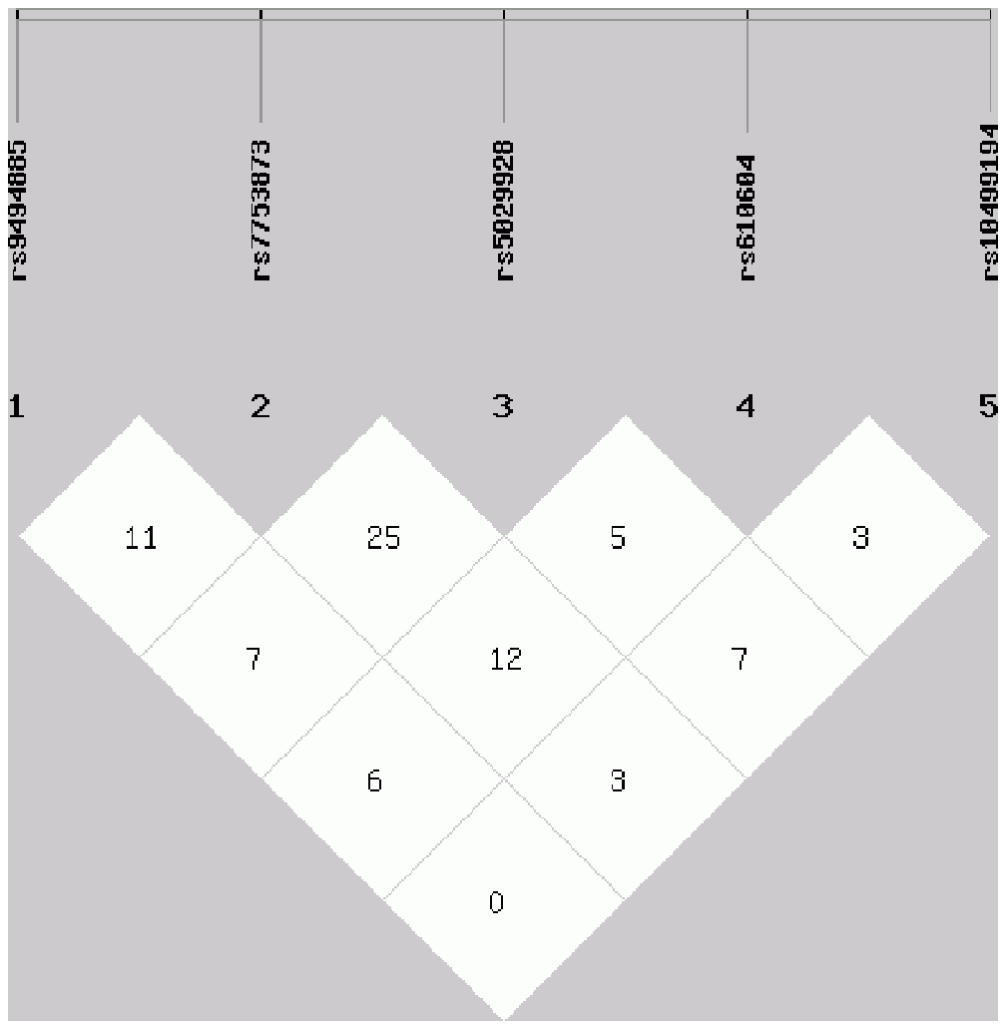
Pair-wised LD result using the data of Guangzhou and Chongqing cohorts. Linkage Disequilibrium (LD) block was estimated for TNFAIP3 gene locus using our data. The pair-wise D’ Values are shown in blocks.

To our knowledge this is the first report on the association between TNFAIP3 gene polymorphisms with VKH disease in a Chinese Han population. In this study we identified one strong risk SNP rs9494885 of TNFAIP3 in Chinese Han VKH patients. A similar association has been reported in SLE in European-ancestry and Korean populations [23]. Although an association has been reported between rs10499194 and RA and SLE in Japanese patients [33], rs7753873and rs5029928 with SLE in European-ancestry and Korean populations [23], and rs610604 with Psoriasis in Europeans [21], we failed to find any association between these SNPs with VKH disease. We recently also studied the association of TNFAIP3 gene polymorphisms with ocular Behcet’s disease (BD) and found that TNFAIP3 was strongly associated with BD in a Chinese Han population. In that study we identified a strong association of rs9494885 (TC genotype: p_c_ = 1.83×10^−10^, TT genotype: p_c_ = 1.23×10^−10^) with BD and two weak associations of rs7753873 (AC genotype: p_c_ = 0.015, AA genotype: p_c_ = 0.03) and rs10499194 (CC genotype: p_c_ = 0.015, TC genotype: p_c_ = 0.015) with this disease [37]. These results seem to suggest that VKH disease has, to a certain extent, similarity in genetic background with some autoimmune diseases and autoinflammatory disease. However, there are also inconsistencies in the association with the various genetic polymorphisms. It is likely that multiple distinct variants of TNFAIP3 could differentially modulate risk of autoimmunity in different diseases and different ancestral backgrounds. Pair-wised LD analysis was also performed using our combined data (Guangzhou and Chongqing cohorts). The result showed that SNPs rs5029928 and rs9494885 are not linked despite of the strong LD in HapMap data (Figure 1). Our controls were collected from Southwest and Southern China, whereas the controls of hapMap came from north China (Beijing Chinese Han), suggesting that there is genetic heterogeneity between the south and the north Han Chinese.

Our previous study [37] showed that SNP rs9494885 did not affect the expression of TNFAIP3, suggesting that the disease associated SNP rs9494885 may be involved in this disease through an unknown mechanism rather than directly regulating TNFAIP3 gene transcription. In addition to mechanisms other than the transcription regulation, SNPs rs9494885 may be in linkage disequilibrium with another causal variant such as copy number variants or indels in this block. More studies are needed to clarify this issue.

It is worthwhile to point out that there are several possible limitations in the present study. The controls and patients enrolled in this study were recruited only from Han Chinese populations. Therefore, the results presented here need to be confirmed in other ethnic cohorts. Additionally, it is not clear whether the examined population size is large enough to detect a sufficient power for the tested SNPs because of a lack of epidemiologic investigations on the prevalence of VKH syndrome in China.

In conclusion, our study showed that the TNFAIP3 SNP rs9494885 was associated with VKH disease in patients of Chinese Han descent and that the rs9494885 C allele and TC genotype may be a risk factor involved in the genetic predisposition to this disease. This association identified in Chinese Han VKH patients is expected to be validated in the study using different ethnic VKH patients.

Additionally a better understanding of how rs9494885 polymorphism in TNFAIP3 affects risk and protection in autoimmunity will provide valuable mechanistic insights into the pathogenesis and treatment of autoimmune disease.
